# Cutaneous Papilloma and Squamous Cell Carcinoma Therapy Utilizing Nanosecond Pulsed Electric Fields (nsPEF)

**DOI:** 10.1371/journal.pone.0043891

**Published:** 2012-08-28

**Authors:** Dong Yin, Wangrong G. Yang, Jack Weissberg, Catherine B. Goff, Weikai Chen, Yoshio Kuwayama, Amanda Leiter, Hongtao Xing, Antonie Meixel, Daria Gaut, Fikret Kirkbir, David Sawcer, P. Thomas Vernier, Jonathan W. Said, Martin A. Gundersen, H. Phillip Koeffler

**Affiliations:** 1 Division of Hematology/Oncology, Cedars-Sinai Medical Center, University of California Los Angeles School of Medicine, Los Angeles, California, United States of America; 2 Alfred E. Mann Institute for Biomedical Engineering, University of Southern California, Los Angeles, California, United States of America; 3 Department of Dermatology, Keck School of Medicine, University of Southern California, Los Angeles, California, United States of America; 4 Ming Hsieh Department of Electrical Engineering, Viterbi School of Engineering, University of Southern California, Los Angeles, California, United States of America; 5 Department of Pathology, University of California Los Angeles School of Medicine, Los Angeles, California, United States of America; 6 Department of Haematology-Oncology, National University Cancer Institute of Singapore, National University Health System, Singapore, Singapore; 7 Cancer Science Institute, Singapore, National University of Singapore, Singapore, Singapore; University of California Berkeley, United States of America

## Abstract

Nanosecond pulsed electric fields (nsPEF) induce apoptotic pathways in human cancer cells. The potential therapeutic effective of nsPEF has been reported in cell lines and in xenograft animal tumor model. The present study investigated the ability of nsPEF to cause cancer cell death *in vivo* using carcinogen-induced animal tumor model, and the pulse duration of nsPEF was only 7 and 14 nano second (ns). An nsPEF generator as a prototype medical device was used in our studies, which is capable of delivering 7–30 nanosecond pulses at various programmable amplitudes and frequencies. Seven cutaneous squamous cell carcinoma cell lines and five other types of cancer cell lines were used to detect the effect of nsPEF *in vitro*. Rate of cell death in these 12 different cancer cell lines was dependent on nsPEF voltage and pulse number. To examine the effect of nsPEF *in vivo*, carcinogen-induced cutaneous papillomas and squamous cell carcinomas in mice were exposed to nsPEF with three pulse numbers (50, 200, and 400 pulses), two nominal electric fields (40 KV/cm and 31 KV/cm), and two pulse durations (7 ns and 14 ns). Carcinogen-induced cutaneous papillomas and squamous carcinomas were eliminated efficiently using one treatment of nsPEF with 14 ns duration pulses (33/39 = 85%), and all remaining lesions were eliminated after a 2nd treatment (6/39 = 15%). 13.5% of carcinogen-induced tumors (5 of 37) were eliminated using 7 ns duration pulses after one treatment of nsPEF. Associated with tumor lysis, expression of the anti-apoptotic proteins Bcl-xl and Bcl-2 were markedly reduced and apoptosis increased (TUNEL assay) after nsPEF treatment. nsPEF efficiently causes cell death *in vitro* and removes papillomas and squamous cell carcinoma *in vivo* from skin of mice. nsPEF has the therapeutic potential to remove human squamous carcinoma.

## Introduction

Nanosecond pulsed electric fields (nsPEF) have been shown to cause cell apoptosis, and investigated as a potential application for cancer therapy [1]. While other cancer therapies, such as chemotherapy and radiotherapy can extensively damage surrounding normal tissues, nanopulse therapy has a very localized effect that can be efficiently delivered solely to the desired site [2]. Nanopulses influence cell activity by a number of means, notably increasing plasma membrane and intracellular membrane permeability and causing alterations of phosphatidylserine distribution (demonstrated by Annexin V binding). Nanopulses also induce intracellular events such as calcium release, caspase activation, and release of cytochrome C into the cytoplasm [1], [3], [4], [5], [6], [7], [8]. Nanopulses can induce apoptosis in human cancer cells [3]. Recently, we found that both tumor and normal skin cells were injured in vitro by nsPEF, and the damage to the tumor cells was greater than damage to the normal cells [9]. Shorter duration nsPEF of up to 20 KV/cm can deliver energy to cells without increasing the temperature of exposed cells for pulse repetition rates of 1 MHz or less [10].

Previous *in vitro* studies have shown that nanopulse therapy inhibits growth of human cancer cells by inducing apoptotic pathways [3], [11]. In solid skin tumors, nanopulses can be directly applied to malignant cells, making nanopulses a viable alternative to surgery for skin cancer patients. Recent studies of melanoma tumors using longer pulses on the order of hundreds of nanoseconds showed that nanopulses stopped blood flow to tumor cells and caused tumor nuclei to shrink. The nanopulses killed melanoma cells without permanently damaging surrounding healthy skin tissue [12], [13], and eliminated the tumors with a single treatment [14]. Full remission resulted after only two treatment sessions (energy of 0.2 J per pulse and 100 pulses delivered with temperature only increasing by 3°C in the localized region) [2].

**Figure 1:**
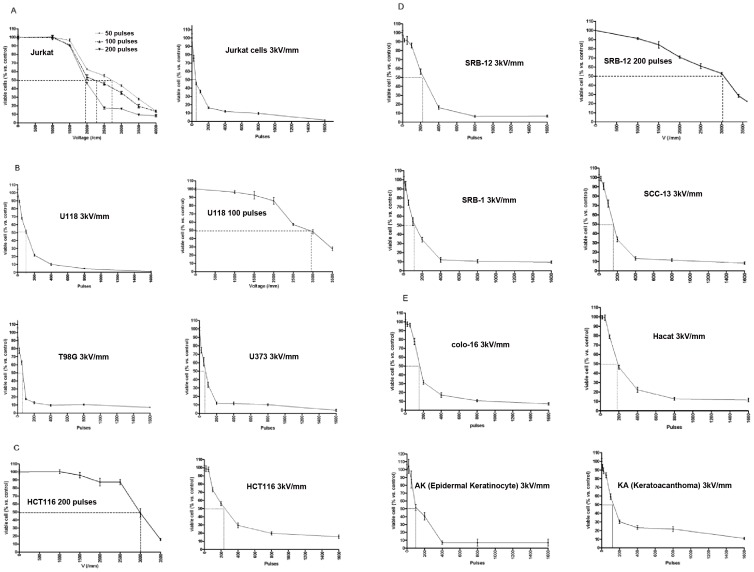
Treatment of cell lines *in vitro* with nsPEF. nsPEF exposure of Jurkat cells (Human T cell leukemia, 2×10^6^/mL) to 50, 100 or 200 pulses of 30 ns duration, at 50 Hz and a varying peak voltage (Panel** A,** left), and at a fixed peak voltage resulting in a field of 30 kV/cm and a varying number of pulses (Panel **A,** right). nsPEF exposure of 11 solid tumor cell lines (2×10^6^/mL in 1 mm cuvette): glioblastoma multiforme (GBM) cells (U118, T98G, U373); colon cancer cells ( HCT116); skin cancer cells (SRB-1, SRB-12, SCC-13, Colo-16, HaCaT); as well as early transformed cells [AK (actinic keratosis), KA (keratoacanthoma)]. Cells were exposed to 100 or 200 pulses of 30 ns duration, at 50 Hz and a varying peak voltage, and at a fixed peak voltage resulting in a field of 30 kV/cm and a varying number of pulses (Panels B. C. D. E). The effect of nsPEF exposure on cell viability is represented by the percentage of viable cells remaining after exposure calculated as a fraction of viable control cells, not exposed but handled similarly. Trypan blue was used to measure viable cells after nsPEF exposure at one hour. The dashed lines indicate either the pulse number or peak voltage associated with a 50% (ED50) reduction in cell viability. Results are values obtained from three experiments under identical conditions (mean + SD).

**Table 1:** Required pulse number at 50, 100, or 200 pulses to kill 50% cells (ED50) of various cancer types.

Cell name	Cell type	Pulses	ED50 (kv/cm)
Jurkat	T-cell leukemia/lymphoma	50	∼27
		100	∼22
		200	∼19
U118	GBM (glioblastoma multiforme)	100	∼29
HCT116	Colon cancer	200	∼30
SRB-12	Skin cancer	200	∼31

**Table 2:** Sensitivity of different cell types to cell death (ED50) caused by nsPEF (30k V/cm).

Cell name	Cell type	ED50 (pulses)
Jurkat	T-cell leukemia/lymphoma	∼50
U118	GBM (glioblastoma multifore)	∼100
T98G	GBM	∼60
U373	GBM	∼70
HCT116	Colon cancer	∼240
AK	Actinic keratosis	∼100
KA	Keratoacanthoma	∼120
SRB-1	Skin cancer	∼100
SRB-12	Skin cancer	∼220
SCC-13	Skin cancer	∼170
Colo-16	Skin cancer	∼170
Hacat	Skin cancer	∼200

**Figure 2:**
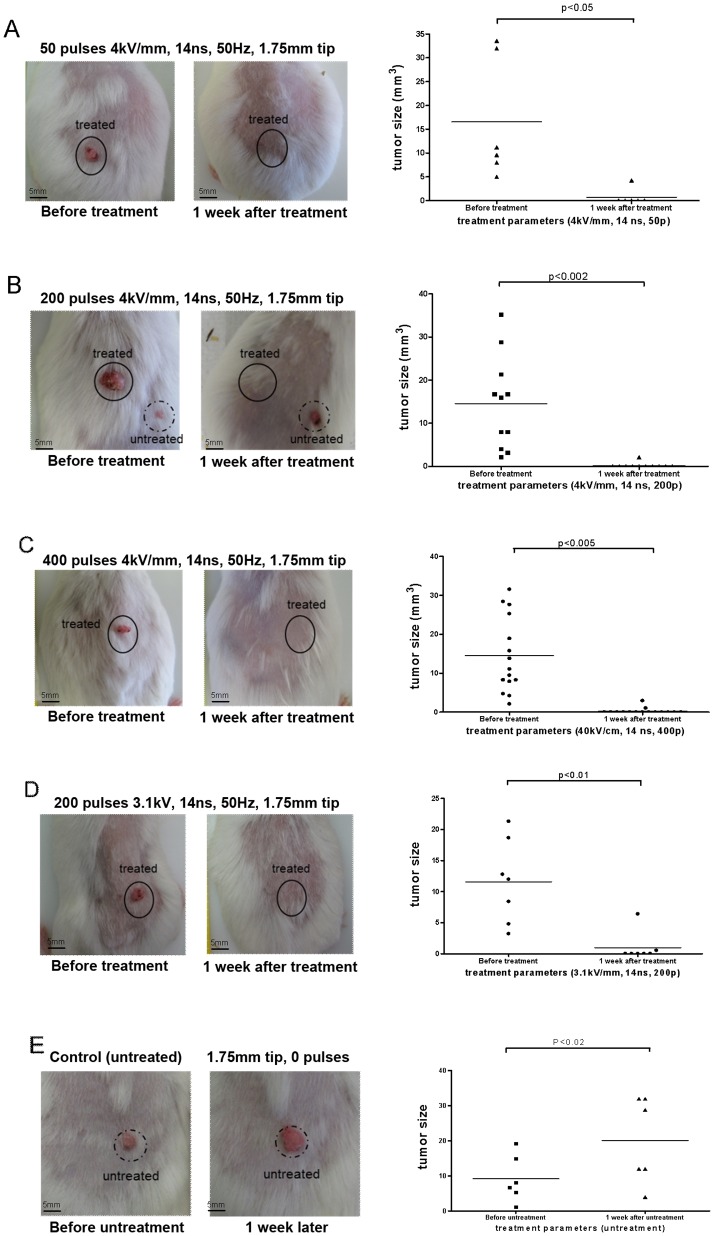
Treatment of induced papillomas and squamous cell carcinomas *in vivo* with nsPEF (14ns). Tumors were exposed to either 50, 200 or 400 pulses of 14 ns duration, 50 Hz and a 40 kV/cm nominal electric field (Panel A, n = 6, median size = 10.4 mm^3^ before treatment; B, n = 11, median size = 15.9 mm^3^ before treatment; C, n = 15, median size = 11.1 mm^3^ before treatment;) and to 200 pulses of 14 ns duration 50 Hz and a 31 kV/cm nominal electric field (Panel D, n = 7, median size = 11.9 mm^3^ before treatment). Unexposed control skin (Panel E, n = 6, median size = 7.3 mm^3^ before untreatment, 20.4 mm^3^ after untreatment). Tumor size was measured prior to exposure and 1-week post exposure. The solid lines depict mean tumor size in each group.

Nanopulse electric fields have the potential to be an effective, minimally invasive treatment for skin tumors. The effect of shorter duration (less than 30 ns) nanopulse exposure of cutaneous squamous carcinoma has not been investigated *in vivo*. To evaluate the responsiveness of cutaneous squamous carcinomas to nanopulse therapy, we applied nanopulses to transformed keratinocyte derived cell lines [HaCaT, Actinic keratosis (AK), Keratoacanthoma (KA)], squamous carcinoma cell lines (SRB-1, SRB-12, SCC-13, and Colo-16), as well as 5 non-skin cancer cell lines *in vitro*. Further, skin papillomas and squamous carcinomas were induced in mice by exposing a patch of their skin to MNNG and TPA. The effectiveness of nanosecond pulsed electric fields *in vivo* against the induced papillomas and cutaneous squamous carcinomas was investigated for first time.

**Figure 3:**
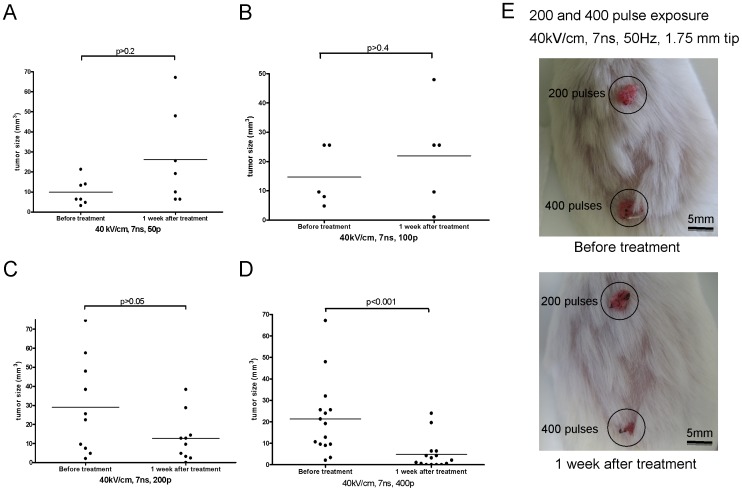
Treatment of induced papillomas and squamous cell carcinomas *in vivo* with nsPEF (7ns). nsPEF exposure (7 ns) of carcinogen induced squamous cell carcinomas *in vivo*. Induced tumors were exposed to 50, 100, 200 or 400 pulses of 7 ns duration, at 50 Hz and 40 kV/cm peak nominal electric field (Panels **A,** n = 7, median size = 6.4 mm^3^ before ureatment, 19.2 mm^3^ after treatment; B, n = 5, median size = 9.6 mm^3^ before ureatment, 25.6 mm^3^ after treatment; C, n = 10, median size = 23.9 mm^3^ before ureatment, 11.2 mm^3^ after treatment; D, n = 15, median size = 19.2 mm^3^ before ureatment, 2.1 mm^3^ after treatment). Tumor sizes were measured prior to and at 1-week post exposure. The solid lines depict mean tumor size in each group. Images (Panel **E**) are representative of the experiments and show pre- and one week post-exposure to either 200 or 400 pulses.

**Table 3:** Effect of nanosecond pulse electric fields on squamous carcinomas *in vivo*.

Experiment	Frequency of pulses (Hz)	Nominal Electric Field (kV/cm)	Number of Pulses per Treatment Site	Pulse Duration (ns)	Carcinomas Eliminated (see Table S1)
1	50	40	50	14	Yes (1 of 6 was retreated)
	50	40	200	14	Yes (1 of 11 was retreated)
	50	40	400	14	Yes (2 of 15 were retreated)
2	50	31	200	14	Yes (2 of 7 were retreated)
3	50	40	50	7	No (n = 7)
	50	40	100	7	No (n = 5)
	50	40	200	7	No (n = 10)
	50	40	400	7	significantly shrunk or eliminated (n = 15)

## Results

### Nanosecond Pulsed Electric Fields Treatment in vitro

Trypan blue was used to assess viable Jurkat human T-cell leukemia/lymphoma cells one hour after nsPEF exposure to determine the effect of electric filed and pulse number on the death of these cells. These cells were exposed to 50, 100 or 200 pulses (30 ns duration at 50 Hz) using various strength of electric field. The percentages of viable cells were calculated compared to unexposed control cells (Figure 1). Cell death caused by nsPEF occurred in a voltage- and pulse number-dependent fashion. For Jurkat cells, the electric field caused 50% cell death (ED50s) following exposure to 50, 100 or 200 pulses at 27 KV/cm, 22 KV/cm and 19 KV/cm, respectively. In all cases (50, 100 and 200 pulses) at an electric field approaching 15 KV/cm, the nsPEF exposure clearly started to cause death of Jurkat cells. At a fixed peak electric field of 30 KV/cm and varying number of pulses, the ED50 was approximately 40 pulses for Jurkat cells (Figure 1 A). In further experiments, the cell viability of the other 11 cell lines was tested, including transformed keratinocyte cell HaCaT, Actinic keratosis (AK), Keratoacanthoma (KA), squamous carcinoma cell lines (SRB-1, SRB-12, SCC-13, and Colo-16), glioblastoma multiforme cells (U118, T98G, U373) and colon cancer cells (HCT116) (Figures 1 B. C. D. E). The ED50s of electric field in 4 cell lines (Jurkat, U118, SRB12, and HCT116) were between 19 KV/cm and 32 KV/cm (Table 1). The ED50 in solid tumor cell lines was approximately 30 KV/cm; therefore, 30 KV/cm was used to examine the pulse numbers that achieved an ED50 in solid tumor cell lines. At 30 KV/cm, these cell lines had ED50s between 60 and 240 pulses (Table 2 and Figure 1).

**Figure 4:**
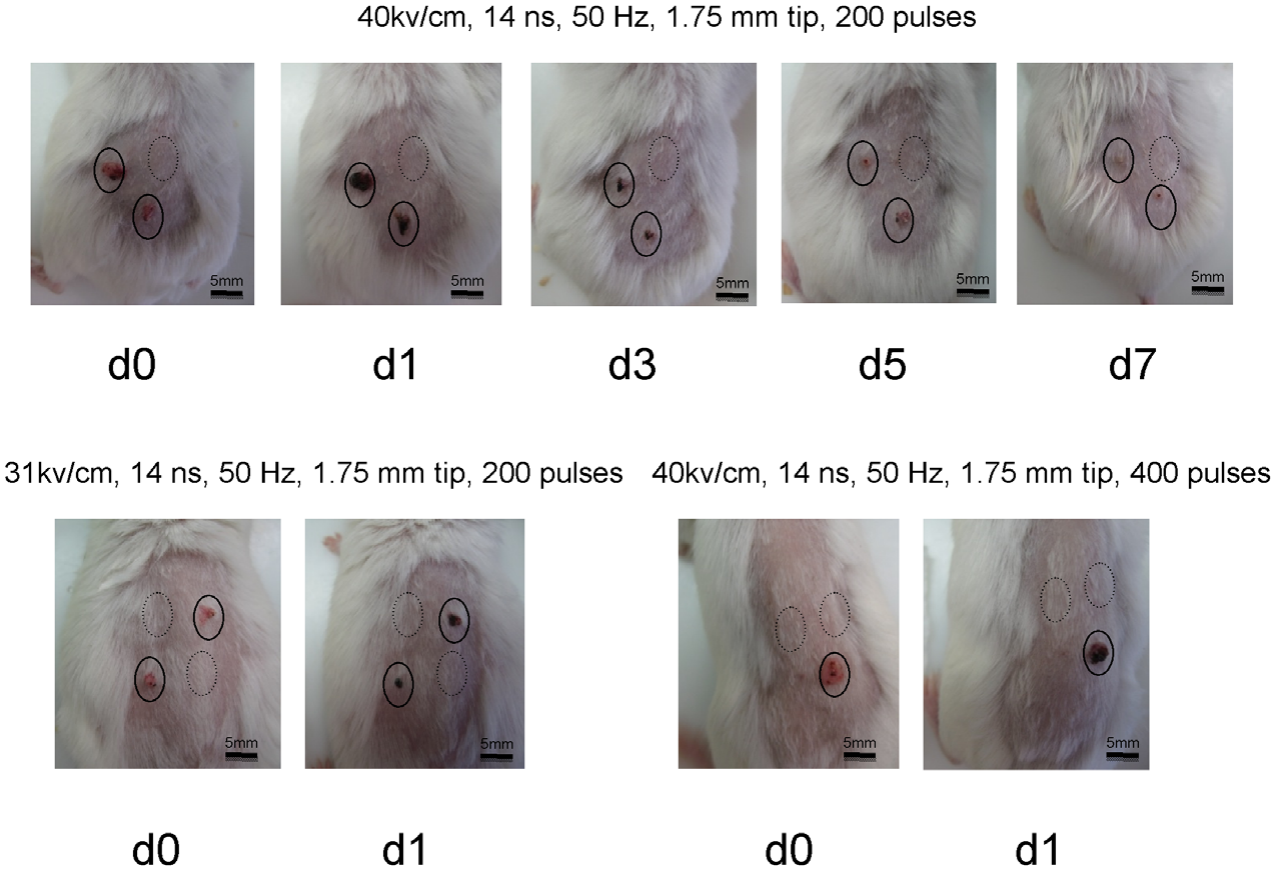
Visual changes over time following nsPEF exposure of induced papillomas and squamous cell carcinomas. Solid circle surround squamous cell carcinomas and dash lines surround normal skin. Appearance at 24 hours (**d1**) post-exposure is shown following nsPEF at 200 and 400 pulses of 14 ns duration, 50 Hz and 40 kV/cm peak nominal electric field and at 200 pulses of 14ns duration, 50 Hz and 31 kV/cm peak nominal electric field. Additional images are shown on alternate days up to one week (**d3**, **d5** and **d7**) for nsPEF exposure of 200 pulses of 14ns duration, 50 Hz and 40 kV/cm nominal electric field.

**Figure 5:**
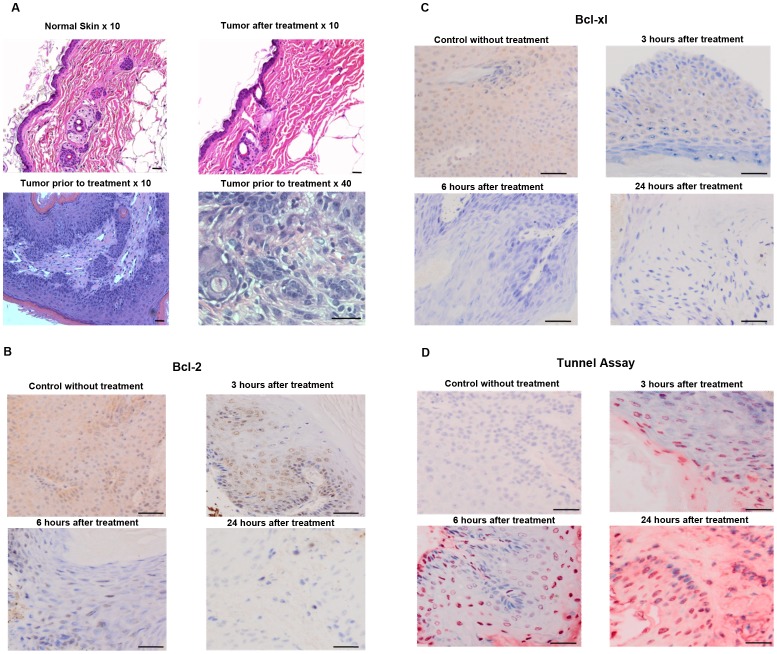
Histology and immunohistochemistry examination. Histopathology (Hematoxylin and Eosin stain) of unaffected normal mouse skin and induced papillomas and squamous cell carcinomas (prior to nsPEF exposure and 5 weeks following effective exposure to 200 pulses of 14 ns duration, 50 Hz, 40 kV/cm) (Panel **A**). Carcinogen induced tumors were treated with nsPEF (40 kV/cm, 50 Hz, 1.75 mm tip 200p). After 3, 6 and 24 hours, immunohistochemistry was performed to detect the anti-apoptotic proteins Bcl-2 (brown in Panel B) and Bcl-xl (brown in Panel C), as well as, apoptosis as shown by TUNEL assay (Terminal deoxynucleotidyl transferase dUTP nick end labeling) (red in Panel D). Scale bar: 50 μm.

### Treatment in vivo with Nanosecond Pulsed Electric Fields

To study the efficiency of nsPEF *in vivo*, a two step carcinogenesis protocol was used (initiator, MMNG; promoter, TPA) to induce papillomas and squamous carcinomas as described in Materials and Methods. After 4–6 months, 2–8 tumors (papillomas and squamous carcinomas) developed on the backs of these mice. In order to examine the ability of nsPEF to eliminate these tumors, three experiments were performed varying different parameters of the nsPEF generator (voltage, pulse duration, and number of pulse). When any dimension of the lesion (length, width or height) was larger than the spacing between the electrodes, multiple sites on each lesion were treated (detail described in Materials and Methods). Based on histology, size and appearance of tumors, about 30% of the treated tumors were squamous cell carcinomas showing signs of invasiveness and 70% were papillomas. For experimental series #1, three different pulse counts were examined at nominal field strength of 40 KV/cm, all at 50 Hz frequency, 14 ns duration, and 1.75 mm tip. papillomas and carcinomas (28/32 = 87.5%) were observed for clearance one week after 50, 200 and 400 pulse treatment using nsPEF (as shown in Figures 2, Table 3 and Table S1). 4 of 32 (12.5%) lesions were reduced in size but not cleared after the first treatment were retreated 1 week following the initial treatment and subsequently cleared. For experimental series #2, the nominal electric field was reduced from 40 KV/cm to 31 KV/cm at 50 Hz (by reducing pulse amplitude from 7KV to 5.5KV), 14 ns duration, 200 pulses, 1.75 mm tip. With this reduced field, 5 out of 7 (71.4%) papillomas and squamous carcinomas were cleared upon observation one week after one treatment (Figure 2 D). The remaining lesions from experiment series #1 and #2 were cleared after a 2^nd^ treatment (6/39 = 15%). In the control experiments, the probe was applied to the tumor without application of nanopulses to deliver a “sham” treatment, showing the effect of the penetrating electrodes without any accompanying electrical energy. Under these conditions, the papillomas and carcinomas continued to grow in each case (Figure 2 E). For experimental series #3, the pulse duration was reduced from 14 ns to 7 ns (40 KV/cm nominal electric field, 50 Hz, 1.75 mm tip). The carcinomas significantly shrunk or disappeared within one week after the first treatment when using 400 pulses, but not after employing 50, 100, or 200 pulses. 13.5% of carcinogen-induced tumors (5 of 37) were eliminated using 7 ns duration pulses after one treatment of nsPEF (Figure 3 and Table S1).

**Figure 6:**
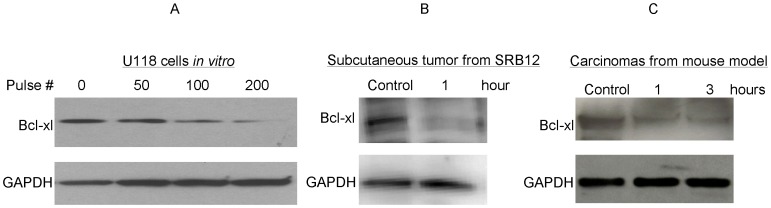
Expression of Bcl-xl *in vitro* and *in vivo*. Western blot analyzed the Bcl-xl expression post nsPEF exposure. Glioblastoma multiforme cell line (U118) was exposed to varying numbers of pulses *in vitro* [20 ns duration, 50 Hz, and 30 kV/cm) in 1 mm cuvette]. 1 hour post nsPEF exposure, Bcl-xl expression was measured (Panel **A**). Squamous carcinoma cell line (SRB-12) was injected subcutaneously into immunocompromised mice. After one week, established tumors were exposed to nsPEF 200 pulses of 14 ns duration, 50 Hz and 40 kV/cm nominal electric field. One hour later, cells were harvested and western blot was performed to measure Bcl-xl expression (Panel **B**). Three induced tumors were either untreated or treated with nsPEF (40 kV/cm nominal electric field, 50 Hz, 1.75 mm tip 200p). Protein was extracted from these tumors after 1 and 3 hours, followed by Western blotted and probed with antibody to Bcl-xl (Panel **C**). GAPDH was used as loading control.

The first day following treatment, the tumors became noticeably darkened, nearly black in places. This dark hue persisted for about 5 days, after which the color faded to pink and then returned to normal color and smoothness. The normal skin, when exposed to the nsPEF, did not visibly show this phenomenon (Figure 4). Figure 5 A shows the histology of these tumors before and after nsPEF treatment. The normal mouse skin has one layer of epidermal squamous cells (upper-left photo). Before treatment, papilloma and/or squamous carcinoma have multiple layers of epidermal squamous cells (lower photos). After treatment, the tumors were eliminated and replaced with a normal layer of epidermal cells (upper-right photo).

**Figure 7:**
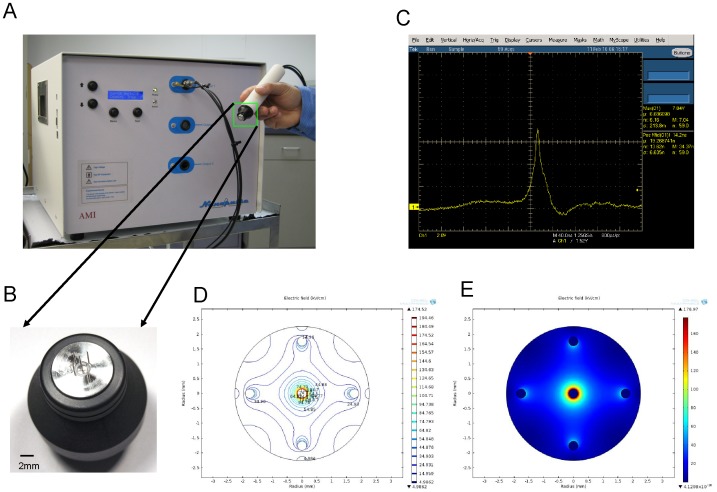
Nanosecond pulsed electric field generator. Image showing the experimental nanosecond pulsed electric field generator and hand piece for delivery of nsPEF exposure to skin tumors in vivo (Panel **A**). Enlarged image showing nsPEF delivery tip; the tip is placed into the tumor during exposure. Significant components of the tip include five short 30-gauge needle electrodes, the center electrode being the positive with four surrounding return electrodes, each spaced 1.75 mm from the center electrode (Panel **B**). Image showing typical pulse waveform, with 7KV amplitude and 14 ns pulse width (FWHM) delivered into a fixed 100 ohm load (Panel **C**).

In a previous study employing similar short (∼10 ns) pulse durations, the nsPEF was shown to cause apoptosis of cells, evident from the caspase release and phosphatidylserine translocation observed [15]. In present study, we found that the nsPEF-treatment of the carcinogen-induced tumors had decreased expression of the apoptosis-protective proteins Bcl-xl and Bcl-2 as measured by immunohistochemistry, and the tumors had increased apoptosis as measured by TUNEL assay (Figures 5 B C and D).

To confirm the immunohistochemistry results, the anti-apoptotic protein Bcl-xl was also examined using western blot. Bcl-xl was markedly reduced within one hour after the cells were treated *in vitro* using nsPEF under 30 KV/cm for either 100 or 200 pulses (Figure 6 A). Because the anti-apoptosis Bcl-xl protein levels in tumor cells decreased *in vitro* after treatment with nsPEF, the expression of this protein was also examined *in vivo*. The first approach was to perform a subcutaneous injection of the human skin cancer cells (SRB12) in immunocompromised mice (Beige Nude XID, NIH III, from Harlan Laboratories) and treat the tumors one week later using nsPEF (nominal 40 KV/cm field, 50 Hz, 1.75 mm tip 200p). After one hour, these tumors were dissected from the mice; the protein was extracted and western-blot analysis was performed. The expression of Bcl-x1 reduced in the treated tumors (Figure 6 B). In addition, carcinogen-induced tumors were treated with nsPEF (nominal 40 KV/cm field, 50 Hz, 1.75 mm tip 200p), and proteins were extracted after 1 and 3 hours. Western-blot analysis showed that Bcl-x1 decreased compared to the mock-treated control cells (Figure 6 C). These results suggest that nsPEF caused apoptosis of the cancer cells both *in vitro* and *in vivo*.

## Discussion

Present studies examined the effects of nano-pulse fields on a variety of cell lines *in vitro* and chemically-induced cutaneous papilloma and squamous cell carcinomas *in vivo*. Previous studies have generally used longer pulse widths (e.g. 60–300 ns) [2], [16]. In contrast, we investigated the effect of shorter (7 to 30 ns), lower energy pulses to determine the energy relationships and other characteristics of this pulse regime.

Our *in vitro* work helped first to identify a relationship between field strength (voltage applied across a fixed distance between electrodes) and induction of cell death. Second, various cell types were examined to determine their sensitivity to the anti-apoptosis effects of nsPEF.

After applying a set number of pulses to Jurkat cells at various voltages using a standard cuvette, the threshold for producing a marked effect was approximately 20 KV/cm, where the onset of effect began at 15 KV/cm. With this threshold established, and along with previous work showing Jurkat cells to be among the more sensitive cell lines to the effects of nanopulse fields [11], 30 KV/cm was chosen as our fixed field strength to use across a variety of cell lines.

ED50s indicated an efficiency of pulses delivered at a fixed-field strength (30 KV/cm) and they were significant differences across cell lines, suggesting that some cells are more sensitive to the nsPEF than other cell types. When examined in aggregate, these differences demonstrated a spread across the cell lines of approximately 4-fold the “total field” required (or total energy applied) in order to produce comparable effects on cell viability.

In experimenting with fairly high fields (50 KV/cm for the Jurkat cell line), and a relatively large number of pulses (400 pulses), a considerable number of fragmented cells were observed at the bottom of the cuvette, indicating that the outer cell membranes of many cells were completely destroyed upon exposure to these fields. This suggests that a necrotic effect on the cellular membranes can be achieved once a large total energy threshold is exceeded. While this is known and expected for longer pulse lengths, such as 100 ns to several ms as used for electroporation applications, this also appears to be the case for short (30 ns) pulses, at least as observed *in vitro*.

Another goal was to understand the relationship between the pulse counts and the field strengths required to destroy carcinomas and pre-cancerous lesions (papillomas) growing in skin, specifically in the epidermal layers of an immunocompetent mouse model. We used a carcinogen-induced skin cancer model in order to be able to test the efficiency of our treatment of skin conditions. Further, using inbred, immunocompetent mice provided a test population which were both healthy and could provide an immune response following the application of pulses, which may contribute to the desired effect of the therapy in clearing the lesions.

The carcinogen-induced skin cancer model produced, as shown by our histopathological analysis, a mix of papillomas and carcinomas, which is consistent with previous studies with this tumor model [17], [18], [19], [20]. After the papillomas and carcinomas resolved, the area was recovered by fur gradually within 1–2 weeks and no further lesion was observed in the same area. These areas were generally observed for an 8 weeks period post treatment. The papillomas occurring in this mouse model are analogous to human hypertrophic actinic keratosis, a condition which is clinically quite common (diagnosed in 14 percent of all visits to dermatologists). Our results demonstrated that this technology is effective on pre-cancerous lesions. Further, the application of the nanopulse therapy was a fast, easy to use treatment modality, with the potential for fewer side-effects and complications versus traditional therapies including surgery.

The *in vivo* studies were designed to understand the number of pulses required reliably to resolve the lesion with a single treatment, as this is the eventual clinical goal for the technology. Using 14 ns pulses, 200 pulses reliably resolved each lesion, with the lesion exposed to at least this number of pulses at multiple sites with the positive electrode applied at approximate 2 mm intervals across its surface (see Materials and Methods for treatment details). As a non-uniform electric field is inherent in the 5 needle arrangement used in these experiments combined with the approximate nature of the electrode placement at multiple sites, it is conceivable that some areas of each lesion treated received more than the prescribed number of pulses (such as 200 pulses of at least 40 KV/cm) intended for each treatment site, and some areas of the surface could have received less if the center electrode was further than 2 mm away from other treatment sites. The algorithm which was adopted for determining the number of treatment sites was designed to account for the non-uniform field and ensure all areas of the lesion were exposed to fields at or above 40 KV/cm, and best efforts were used to evenly space the treatment sites and ensure coverage of the entire lesion. The clearance results achieved in these experiments suggests these methods were effective.

For a given field strength, pulse duration and electrode arrangement, a threshold appeared in terms of pulse count. Once past this threshold, the tumors generally resolved in a single treatment; and below the threshold, the lesions shrank or disappeared less reliably and may require multiple treatments to resolve completely. At shorter pulses (7 ns), a threshold of 400 pulses was required to eliminate lesions with a single treatment, with similar overall results as compared to the 14 ns pulses once this threshold was reached, but requiring over 4 times the “total field” applied for a pulse duration half as long. This suggests a non-linear, “square” relationship between total energy (or the number of pulses) applied and pulse duration, and this confirms findings theorized and suggested in previous studies [2].

Control tumors in multiple mice continued to grow in spite of being penetrated with the 5 needle electrode tips in multiple sites in a single tumor yet with no pulses delivered. While they may initially have shrunk slightly due to the mechanical effects of the penetrating needles, the tumors recovered and began to grow within several days after the contact with the needle electrodes.

Areas of normal skin were also exposed to nsPEF, to observe the effects of these short duration pulses qualitatively over the days and weeks following treatment. Effects on normal skin were resolved within 1 week and no significant scarring or lasting effects were noted. In a separate *in vitro* study, comparing paired cancer to normal skin cell lines after treated by nsPEF, both cancer cells and normal cells could be injured by nsPEF, but the damage was more extensive in the cancer cells and the recovery was quicker in the normal cells [9]. The results of studies in vitro suggested that the normal mouse skin cell could also be affected by nsPEF and recover quickly *in vivo*.

Significantly, we were able to achieve complete clearance of tumors and papillomas with a single treatment using nsPEF. This can presumably lead to less collateral damage to surrounding healthy tissue, reduced scarring, allow faster healing, and permit the use of more efficient, more reliable and smaller devices for clinical applications. Further, the use of fewer, shorter pulses leads to reduced treatment time and increased physician productivity, both valuable benefits which can aid adoption of a new treatment paradigm such as nanosecond pulsed electric fields.

## Materials and Methods

### Cell culture

Squamous skin carcinoma cell lines were obtained from American Type Culture (ATCC) or provided generously by Dr. Reuben Lotan (The University of Texas, M. D. Anderson Cancer Center) and maintained in K-SFM + supplements (Invitrogen 10724-011) +3% fetal bovine serum (FBS, GIBCO, Carlsbad, CA). The other cell lines (from ATCC) were maintained in DMEM growth media with 10% FBS and 1% penicillin/streptomycin (GIBCO, Carlsbad, CA). All cells were grown in a 37°C, 5% CO_2_ tissue culture incubator.

### Pulsed electric field application on cells in vitro

Monolayer cells were trypsinized, washed, pelleted and resuspended at 1×10^6^ cells/ml of DMEM growth media with 10% FBS. Cell suspensions were exposed to electric pulses in commercial electroporation cuvettes (Genetronics, Inc., San Diego, CA) with 1 mm electrode spacing. The pulse generator was a solid-state, resonant-charged, magnetic compression, diode-opening switch-based system designed and fabricated at the University of Southern California specifically for testing these short pulses with in vitro loads. The device delivers 30 ns, 30 KV/cm pulses at repetition rates of 50 Hz to low-impedance (10–20 ohm) loads. After pulsed electric field exposure, cells were immediately diluted to 5×10^5^ cells/ ml and incubated in a 37°C, 5% CO_2_ tissue culture incubator. After 60 minutes of incubation, cells were stained using typan blue, and viable cells were counted using a light microscope.

### Mouse model of induced skin squamous carcinoma

Cutaneous squamous carcinomas were induced in SENCAR (SENsitivity to CARcinogenesis) and CD-1 mice. SENCAR mice were developed from CD-1 mice by recurrent selection of mice that are sensitive to chemical induced tumor development [14], [15] and were generously given to us by Linda Blumenauer (NCI-Frederick). CD-1 mice were bought from Charles River. Both SENCAR and CD-1 mice were maintained in Cedars Sinai Medical Center's animal facility. Cutaneous papillomas and squamous carcinomas were chemically induced according to an established protocol [14], [15], [16]. Carcinogen was applied on the flank of the shaven murine skin using a cotton-tipped applicator. Briefly, tumors were initiated using methyl-N'-nitro-N-nitrosoguanidine (MNNG, 2 μmol) on the first week followed by promotion of the tumor using 12-O-tetradecanoylphorbol-13-acetate (TPA, 2ug) applied weekly. After 20–30 weeks, papillomas or carcinomas were detected visually and characterized by rapid growth with elevated margins. Morphology of induced tumors was periodically examined using standard histology. The sizes of the tumors were recorded and photographs were taken before and after each nsPEF treatment.

### Nanosecond pulsed electric field application on cutaneous papillomas and squamous carcinoma in vivo

The pulse generator was a prototype medical device (Figure 7), produced by the Alfred E. Mann Institute for Biomedical Engineering at the University of Southern California, which is capable of selectively delivering either 7 or 14 nanosecond duration pulses (measured at full width, half maximum) at various programmable amplitudes and frequencies. An applicator tip consisting of five 30 gauge needles, each 5 mm long, in a configuration with 1 positive electrode surrounded by 4 negative electrodes was used for all experiments (Figure 7 B). The spacing between the positive and negative electrodes was 1.75 mm. With this electrode configuration, the resulting electrical field is non-uniform, and this is illustrated in Figure 7 (D and E) which indicates the field strengths generated across a horizontal slice of the volume of tissue treated at the peak pulse amplitude of 7KV. For simplicity, we refer to the field strengths at various pulse amplitudes by their “nominal” values, which are obtained from the peak pulse amplitude divided by the spacing between the electrodes. To avoid the occurrence of air pockets between the electrodes, both tumor and tip were covered with Aquasonic 100 ultrasound transmission gel (Parker Laboratories, Inc. Fairfield, NJ., USA). A frequency (or repetition rate) of 50 Hz was chosen to standardize upon for the in vivo experiments as it provided reasonable treatment times of several seconds per exposure site for the pulse counts investigated, and matched the frequency used for the in vitro work performed in this study. Two pulse durations (14 ns and 7 ns) were used for the experiments with varying pulse numbers and amplitude to determine the effect of varying these parameters on tumor clearance (Figure 7 C).

The length, width and height of each lesion were measured. The volume of lesion is 0.5236× length × width × height. When any dimension of lesion was larger than the spacing between the electrodes (1.75 mm), the pulses were applied to multiple treatment sites on the surface of the lesion, spaced approximately 2 mm apart. This was done to ensure the entire tumor surface was exposed to the nominal field of 40 KV/cm, which surrounds the center electrode with a radius of 1 mm. The number of treatment sites per lesion was determined according to the largest dimension of the lesion. For example, when using 200 pulses, the lesion was treated in 2 treatment sites for the 2 mm lesion (longest dimension) with 200 pulses applied at each treatment site, 3 treatment sites for the 3 mm lesion with 200 pulses applied at each treatment site, 4 treatment sites for the 4 mm length lesion, etc. The treatment sites were distributed approximately evenly across the lesion surface to ensure complete coverage of each lesion, with the center positive electrode penetrating the lesion surface at each treatment site. Any lesion smaller than 2 mm×2 mm×2 mm was treated at just one treatment site, approximately in the center of the lesion. Lesions still present after the initial treatment and requiring a 2^nd^ treatment were re-treated 1 week following the initial treatment. The algorithm described above for determining the number of treatment sites per lesion was used for those tumors requiring a 2^nd^ treatment, as well, based on the largest dimension of the lesion at the time of re-treatment. Tumors were considered cleared (eliminated) when no recurrence was detected within 4–8 weeks after the last treatment.

Mice were given isofluorane anesthesia and positioned on a warming bed for all procedures, each of which was approved by the IRB of Cedars Sinai Medical Center Comparative Medicine (IACUC002427).

### Histology/Immunohistology

After mice had complete tumor regression in areas exposed to nsPEF therapy for at least four weeks, skin samples were taken and fixed for histology. The skin from tumor locations which had been exposed to nsPEF therapy and had cleared, the skin from control tumors which were penetrated by the applicator tip but not exposed to nsPEF therapy, as well as a sample of normal skin were excised and immediately placed in a 10% buffered formalin solution for 24 hours at room temperature and then transferred into 70% ethanol. After paraffin embedding, 5 μm sections were cut every 0.5 mm across each skin sample and stained with H&E. Stained slides were observed using microscopy.

The carcinogen-induced tumors were dissected before and after nsPEF treatment and untreated tumors were dissected and examined for their expression of anti-apoptosis protein Bcl-xl and Bcl-2 using immunohistochemistry as previously described [17]. Rabbit monoclonal antibodies to Bcl-2 and Bcl-xl (Abcam) were applied at a dilution of 1∶50 for 60 min at room temperature. The sections were counterstained with hematoxylin. Tunnel assay was also performed using a kit from Roche (Cat. No. 11684809910).

### Western blot analysis

The samples were collected after nsPEF treatment in vitro and in vivo. Western blot was performed as previously described [17]. All antibodies were bought from Santa Cruz Biotechnology Inc. (California, USA).

### Statistical Analysis

Differences between the results of experimental treatments were evaluated by a t-test. Differences were considered significant at values of p<0.05.

## Supporting Information

Table S1 **Lesion and Treatment Detail.**
(DOCX)
