# Supplementary material for: Cutaneous Papilloma and Squamous Cell Carcinoma Therapy Utilizing Nanosecond Pulsed Electric Fields (nsPEF)
Source: PLoS One. 2012 Aug 28;7(8):e43891. doi: 10.1371/journal.pone.0043891 (PMC3429422; doi:10.1371/journal.pone.0043891)
Supplement: Table S1 — Lesion and Treatment Detail. (DOCX) [file pone.0043891.s001.docx]

**Table S1: Lesion and Treatment Detail**

| Experiment | Size  (Before treatment) | | | | Treatment  sites per lesion | Size  (1 week after treatment) | | | |
| --- | --- | --- | --- | --- | --- | --- | --- | --- | --- |
|  | Length  (mm) | Width  (mm) | Height  (mm) | Volume  (mm^3^) |  | Length  (mm) | Width  (mm) | Height  (mm) | Volume  (mm^3^) |
| 40 kV/cm, 14ns, 50p  (Lesions in Figure 2 A) | 3.5 | 3 | 2 | 11.18 | 4 | 0 | 0 | 0 | 0.0 |
|  | 4 | 3 | 1.5 | 9.59 | 4 | 0 | 0 | 0 | 0.0 |
|  | 2.5 | 2.5 | 1.5 | 4.99 | 3 | 2 | 2 | 2 | 4.26* |
|  | 3 | 2.5 | 2 | 7.99 | 3 | 0 | 0 | 0 | 0.0 |
|  | 4.5 | 4 | 3.5 | 33.55 | 5 | 0 | 0 | 0 | 0.0 |
|  | 5 | 4 | 3 | 31.96 | 5 | 0 | 0 | 0 | 0.0 |
| 40kV/cm, 14 ns, 200p (Lesions in Figure 2 B) | 5 | 4 | 2 | 21.3 | 5 | 0 | 0 | 0 | 0.0 |
|  | 2 | 2 | 1 | 2.13 | 2 | 0 | 0 | 0 | 0.0 |
|  | 4 | 3 | 2.5 | 15.98 | 4 | 0 | 0 | 0 | 0.0 |
|  | 6 | 3 | 3 | 28.76 | 6 | 0 | 0 | 0 | 0.0 |
|  | 3 | 2.5 | 2 | 7.99 | 3 | 0 | 0 | 0 | 0.0 |
|  | 4.5 | 3.5 | 2 | 16.78 | 5 | 0 | 0 | 0 | 0.0 |
|  | 5.5 | 4 | 3 | 35.15 | 6 | 0 | 0 | 0 | 0.0 |
|  | 4.5 | 3.5 | 2 | 16.8 | 5 | 0 | 0 | 0 | 0.0 |
|  | 2 | 2 | 1.5 | 3.2 | 2 | 0 | 0 | 0 | 0.0 |
|  | 2.5 | 3 | 2 | 7.99 | 3 | 0 | 0 | 0 | 0.0 |
|  | 3 | 2.5 | 1 | 3.99 | 3 | 2 | 2 | 1 | 2.13* |
| 40 kV/cm, 14ns, 400p (Lesions in Figure 2 C) | 4 | 3 | 3 | 19.17 | 4 | 0 | 0 | 0 | 0.0 |
|  | 6 | 4 | 2 | 25.56 | 6 | 0 | 0 | 0 | 0.0 |
|  | 5 | 4 | 4 | 42.61 | 5 | 0 | 0 | 0 | 0.0 |
|  | 3 | 1.5 | 2 | 4.79 | 3 | 0 | 0 | 0 | 0.0 |
|  | 3 | 2.5 | 2 | 7.99 | 3 | 0 | 0 | 0 | 0.0 |
|  | 3.5 | 3 | 1.5 | 8.39 | 4 | 0 | 0 | 0 | 0.0 |
|  | 2 | 2 | 1 | 2.13 | 2 | 2 | 1 | 1 | 1.07* |
|  | 5 | 3.5 | 3 | 27.96 | 5 | 0 | 0 | 0 | 0.0 |
|  | 3.5 | 3 | 2 | 11.18 | 4 | 0 | 0 | 0 | 0.0 |
|  | 3.5 | 2.5 | 3 | 13.98 | 4 | 2.5 | 1.5 | 1.5 | 2.99* |
|  | 3.5 | 3 | 1.5 | 8.39 | 4 | 0 | 0 | 0 | 0.0 |
|  | 5 | 4 | 3 | 31.96 | 5 | 0 | 0 | 0 | 0.0 |
|  | 4.5 | 4 | 3 | 28.76 | 5 | 0 | 0 | 0 | 0.0 |
|  | 4 | 3 | 2.5 | 15.98 | 4 | 0 | 0 | 0 | 0.0 |
|  | 3 | 3 | 2 | 9.59 | 3 | 0 | 0 | 0 | 0.0 |
| 31 kV/cm, 14ns, 200p (Lesions in Figure 2 D) | 5 | 4 | 2 | 21.30 | 5 | 0 | 0 | 0 | 0.0 |
|  | 3.5 | 3 | 1.5 | 8.39 | 4 | 1 | 1 | 1 | 0.53* |
|  | 4 | 3.5 | 2.5 | 18.64 | 4 | 0 | 0 | 0 | 0.0 |
|  | 3 | 3 | 2.5 | 11.98 | 3 | 0 | 0 | 0 | 0.0 |
|  | 6 | 3 | 3 | 12.78 | 6 | 3 | 2 | 2 | 6.39* |
|  | 3 | 3 | 1 | 4.79 | 3 | 0 | 0 | 0 | 0.0 |
|  | 4 | 3 | 0.5 | 3.2 | 4 | 0 | 0 | 0 | 0.0 |
| Sham  Treatment (Lesions in Figure 2 E) | 2.5 | 2.5 | 2 | 6.66 | 3 | 2.5 | 3 | 3 | 11.98 |
|  | 2 | 1 | 1 | 1.07 | 2 | 2 | 1.5 | 2.5 | 3.99 |
|  | 3 | 2.5 | 2 | 7.99 | 3 | 6 | 3 | 3 | 28.76 |
|  | 2.5 | 2 | 2 | 5.33 | 3 | 3 | 3 | 2.5 | 11.98 |
|  | 4 | 2 | 3.5 | 14.91 | 4 | 4 | 5 | 3 | 31.96 |
|  | 4 | 3 | 3 | 19.17 | 4 | 4 | 5 | 3 | 31.96 |
| 40 kV/cm, 7ns, 50p (Lesions in Figure 3 A) | 3 | 2 | 2 | 6.39 | 3 | 3 | 2 | 2 | 6.39 |
|  | 3 | 2 | 2 | 6.39 | 3 | 3 | 2 | 2 | 6.39 |
|  | 3.5 | 2.5 | 3 | 13.98 | 4 | 3 | 2.5 | 2.5 | 9.99 |
|  | 3 | 2 | 1.5 | 4.79 | 3 | 6 | 5 | 3 | 47.93 |
|  | 5 | 5 | 1 | 13.31 | 5 | 7 | 6 | 3 | 67.11 |
|  | 3 | 2 | 1 | 3.19 | 3 | 6 | 4 | 2 | 25.57 |
|  | 5 | 4 | 2 | 21.30 | 5 | 4 | 3 | 3 | 19.17 |
| 40kV/cm, 7ns, 100p (Lesions in Figure 3 B) | 3 | 2.5 | 2 | 7.99 | 3 | 6 | 5 | 3 | 47.93 |
|  | 3 | 3 | 2 | 9.59 | 3 | 2 | 1 | 1 | 1.07 |
|  | 6 | 4 | 2 | 25.56 | 6 | 6 | 4 | 2 | 25.56 |
|  | 6 | 4 | 2 | 25.56 | 6 | 6 | 4 | 2 | 25.56 |
|  | 3 | 3 | 1 | 4.79 | 3 | 3 | 3 | 2 | 9.59 |
| 40kV/cm, 7ns, 200p (Lesions in Figure 3 C) | 3 | 3 | 1 | 4.79 | 3 | 3 | 3 | 1 | 4.79 |
|  | 3 | 2 | 3 | 9.59 | 3 | 3 | 3 | 0.5 | 2.39 |
|  | 4 | 3.5 | 1 | 7.46 | 4 | 4 | 3 | 2 | 12.78 |
|  | 7 | 5 | 4 | 74.56 | 7 | 4 | 3 | 0.5 | 3.19 |
|  | 6 | 6 | 3 | 57.52 | 6 | 6 | 4 | 3 | 38.35 |
|  | 2 | 2 | 1 | 2.13 | 2 | 3 | 3 | 2 | 9.59 |
|  | 4 | 3.5 | 3 | 22.37 | 4 | 3 | 3 | 3 | 14.38 |
|  | 6 | 5 | 3 | 47.93 | 6 | 6 | 4.5 | 2 | 28.76 |
|  | 6 | 4 | 2 | 25.56 | 6 | 4 | 3 | 2 | 12.78 |
|  | 6 | 4 | 3 | 38.35 | 6 | 0 | 0 | 0 | 0.0 |
| 40kV/cm, 7ns, 400p (Lesions in Figure 3 D) | 5 | 4 | 2 | 21.30 | 5 | 3 | 2 | 1 | 3.19 |
|  | 2 | 2 | 1 | 2.13 | 2 | 2 | 2 | 0.5 | 1.07 |
|  | 2.5 | 2.5 | 1 | 3.33 | 3 | 2 | 2 | 1 | 2.13 |
|  | 6 | 5 | 3 | 47.93 | 6 | 1 | 1 | 1 | 0.53 |
|  | 3 | 3 | 2 | 9.59 | 3 | 0 | 0 | 0 | 0.0 |
|  | 4 | 2 | 2 | 8.96 | 4 | 3 | 2 | 2 | 6.39 |
|  | 5 | 4 | 3 | 31.96 | 5 | 0 | 0 | 0 | 0.0 |
|  | 7 | 6 | 3 | 67.11 | 7 | 5 | 3 | 3 | 23.97 |
|  | 6 | 3 | 2.5 | 23.97 | 6 | 1 | 1 | 1 | 0.53 |
|  | 4 | 4 | 3 | 25.56 | 4 | 3.5 | 3.5 | 3 | 19.57 |
|  | 6 | 4 | 2 | 25.56 | 6 | 4 | 2 | 1 | 4.26 |
|  | 4 | 3 | 2 | 12.78 | 4 | 0 | 0 | 0 | 0.0 |
|  | 4 | 3 | 3 | 19.17 | 4 | 4 | 3 | 1 | 6.39 |
|  | 3 | 3 | 2 | 9.59 | 3 | 4 | 2 | 1 | 4.26 |
|  | 4 | 2.5 | 2 | 10.65 | 4 | 0 | 0 | 0 | 0.0 |

|  |
| --- |

Note: * indicates the tumor was treated a second time 1 weeks following the initial treatment and was subsequently cleared.
